# Noninferiority Margin Size and Acceptance of Trial Results: Contingent Valuation Survey of Clinician Preferences for Noninferior Mortality

**DOI:** 10.1177/0272989X221099493

**Published:** 2022-05-18

**Authors:** Sandra Pong, Robert A. Fowler, Nicholas Mitsakakis, Srinivas Murthy, Jeffrey M. Pernica, Elaine Gilfoyle, Asha Bowen, Patricia Fontela, Winnie Seto, Michelle Science, James S. Hutchison, Philippe Jouvet, Asgar Rishu, Nick Daneman

**Affiliations:** Department of Pharmacy, The Hospital for Sick Children, Toronto, ON, Canada; Interdepartmental Division of Critical Care Medicine, University of Toronto, Toronto ON, Canada; Tory Trauma Program, Sunnybrook Health Sciences Centre, Toronto, ON, Canada; Institute of Health Policy, Management and Evaluation, University of Toronto, Toronto, ON, Canada; Children’s Hospital of Eastern Ontario Research Institute, Ottawa, ON, Canada; Dalla Lana School of Public Health, University of Toronto, Toronto, ON, Canada; Department of Pediatrics, Division of Critical Care, University of British Columbia, Vancouver, BC, Canada; Research Institute, BC Children’s Hospital, Vancouver, BC, Canada; Division of Infectious Diseases, McMaster University, Hamilton, ON, Canada; Department of Critical Care Medicine, The Hospital for Sick Children, Toronto, ON, Canada; Wesfarmers Centre for Vaccines and Infectious Diseases, Telethon Kids Institute, University of Western Australia Perth Children’s Hospital, Nedlands, WA, Australia; Department of Infectious Diseases, Perth Children’s Hospital, Nedlands, WA, Australia; Department of Epidemiology, Biostatistics and Occupational Health, McGill University, Montreal, QC, Canada; Department of Pediatrics, McGill University, Montreal, QC, Canada; Department of Pharmacy, The Hospital for Sick Children, Toronto, ON, Canada; Institute of Health Policy, Management and Evaluation, University of Toronto, Toronto, ON, Canada; Leslie Dan Faculty of Pharmacy, University of Toronto, Toronto, ON, Canada; Division of Infectious Diseases, Department of Paediatric Medicine, The Hospital for Children, Toronto, ON, Canada; Department of Critical Care Medicine, The Hospital for Sick Children, Toronto, ON, Canada; Pediatric Intensive Care Unit, Sainte-Justine Hospital University Center, Montreal, QC, Canada; Department of Pediatrics, Université de Montréal, Montreal, QC, Canada; Critical Care Research Unit, Sunnybrook Health Sciences Centre, Toronto, ON, Canada; Division of Infectious Diseases, Sunnybrook Health Sciences Centre, Toronto, ON, Canada

**Keywords:** antimicrobials, contingent valuation, duration of therapy, evidence uptake, mortality, noninferiority margin

## Abstract

**Objectives:**

We used modified contingent valuation methodology to determine how noninferiority margin sizes influence clinicians’ willingness to accept clinical trial results that compare mortality in critically ill children.

**Methods:**

We surveyed pediatric infectious diseases and critical care clinicians in Canada, Australia, and New Zealand and randomized respondents to review 1 of 9 mock abstracts describing a noninferiority trial of bacteremic critically ill children assigned to 7 or 14 d of antibiotics. Each scenario showed higher mortality in the 7-d group but met noninferiority criterion. We explored how noninferiority margins and baseline mortality rates influenced respondent acceptance of results.

**Results:**

There were 106 survey respondents: 65 (61%) critical care clinicians, 28 (26%) infectious diseases physicians, and 13 (12%) pharmacists. When noninferiority margins were 5% and 10%, 73% (24/33) and 79% (27/33) respondents would accept shorter treatment, compared with 44% (17/39) when the margin was 20% (*P* = 0.003). Logistic regression adjusted for baseline mortality showed 5% and 10% noninferiority margins were more likely to be associated with acceptance of shorter treatment compared with 20% margins (odds ratio [OR] 3.5, 95% confidence interval [CI]: 1.3–9.6, *P* = 0.013; OR 5.1, 95% CI: 1.8–14.6, *P* = 0.002). Baseline mortality was not a significant predictor of acceptance of shorter treatment.

**Conclusions:**

Clinicians are more likely to accept shorter treatment when noninferiority margins are ≤10%. However, nearly half of respondents who reviewed abstracts with 20% margins were still willing to accept shorter treatment. This is a novel application of contingent valuation methodology to elicit acceptance of research results among end users of the medical literature.

**Highlights:**

- Clinicians are more likely to accept shorter treatment durations based on noninferior mortality results when the noninferiority margin is 5% or 10% than if the margin is 20%.
- However, nearly half of clinicians would still accept shorter-duration treatment as noninferior with margins of 20%.
- Baseline mortality does not independently predict acceptance of shorter-duration treatment.
- Contingent valuation is a novel approach to elicit the acceptance of research design parameters from the perspective of endusers of the medical literature.

## Introduction

Noninferiority studies aim to demonstrate that a new treatment is no worse than a standard intervention by a prespecified noninferiority margin chosen by researchers. As there are no firmly established norms, the acceptable width of the margin is a controversial aspect of study design and critically important because it is a determinant of sample size and helps to frame the interpretation of results.^1,2^ A systematic review of noninferiority trials of medications with primary outcomes involving mortality showed an overall median absolute noninferiority margin of 9% (interquartile range 4.2%–10%).^3^ However, prior research has not yet examined how noninferiority margin sizes affect the acceptance of study results by users of the medical literature. These considerations are important when designing research and anticipating how clinical trial outcomes could be interpreted and eventually influence clinical practice.

Contingent valuation is a methodology that elicits an individual’s valuation of benefits of a commodity that is not available in the market.^4,5^ We adapted contingent valuation methodology as a novel means to determine how the size of noninferiority margins could influence clinicians’ willingness to accept noninferior mortality in a hypothetical trial comparing shorter versus longer antibiotic treatment duration in critically ill children with bacteremia. We chose mortality as a robust and objective primary outcome measure because it is undesirable from all researcher, clinician, and patient perspectives. Insight into plausible estimates of noninferiority margin sizes that could be considered acceptable among clinicians may help inform decisions about sample size requirements in future noninferiority trials conducted in children, guide research design decisions that incorporate perspectives of end users of the medical literature, and maximize the impact of study results in pediatric clinical practice.

## Methods

### Study Population

We conducted this study in conjunction with a bacteremia antibiotic treatment duration survey among pediatric infectious diseases (ID) and critical care clinicians in Canada, Australia, and New Zealand in winter 2020–2021.^6^ Critical care clinicians and pharmacists in Canadian pediatric intensive care units were contacted by email with invitations to participate in the anonymous, online web-based survey via SurveyMonkey. The ID clinicians surveyed belonged to the Paediatric Investigators Collaborative Network on Infections in Canada, and the Australia and New Zealand Paediatric Infectious Diseases Group (ANZPID) of the Australasian Society of Infectious Diseases (ASID). Research Ethics Board approval was granted at Sunnybrook Health Sciences Centre, University of Toronto.

### Survey Design and Outcomes

We randomized consenting respondents to review 1 of 9 abstracts of a hypothetical noninferiority trial of bacteremic critically ill children who were randomly assigned to either 7 or 14 d of antibiotics in which the primary outcome was mortality. Each mock abstract specified a noninferiority margin, point estimate, and 95% confidence interval of the difference in mortality between the 7- and 14-d treatment groups. We modified these parameters with noninferiority margins set at 5%, 10%, or 20% and baseline mortality rates in the 14-d control group at 5%, 10%, or 15%. In every scenario, the point estimate for mortality was higher in the 7-d group, but the noninferiority criterion was met based on the margin (Supplemental Table S1). We asked survey respondents whether they would accept the study results presented to them as a justification of shortening the treatment duration of bacteremia to 7 d in critically ill children.

Seven clinicians (critical care physicians, ID physicians, pharmacist, nurse practitioner) pilot tested the abstract to assess flow, acceptability, ease of administration, and clarity.^7^

### Sample Size

A target of 97 respondents allowed for a 95% two-sided confidence interval to extend ±10% around an expected 50% of respondents who would accept trial results if the absolute noninferiority margin was ≤10% (α = 0.05).

### Statistical Analyses

For each scenario, we calculated the proportion of respondents who would accept noninferior mortality results to justify a shorter duration of antibiotic therapy. We performed chi-square or Fisher's exact tests to determine if there was a relationship between the acceptance of trial results at different levels of noninferiority margins and baseline mortality rates and clinician practice specialty. We also evaluated the relationship between the acceptance of trial results and noninferiority margins using logistic regression, adjusted for baseline mortality rates. Statistical analyses were conducted using SAS software version 9.4M6 (SAS Institute, Cary, NC). This study was not funded.

## Results

There were 106 survey respondents: 65 (61%) critical care clinicians, 28 (26%) ID physicians, and 13 (12%) intensive care– or ID-focused pharmacists.^6^ Respondents had a broad range of clinical experience: ≤5 y (6%), 6 to 10 y (22%), 11 to 15 y (24%), 16 to 20 y (23%), and ≥21 y (26%).

When noninferiority margins for mortality in the abstracts were 5% and 10%, a shorter duration of antibiotic treatment was accepted by 73% and 79% of respondents, respectively. Acceptance was lower at 44% when the noninferiority margin was 20% (*P* = 0.003; Table 1).

**Table 1:** Proportion of Respondents Accepting or Rejecting Shorter-Duration Treatment According to Noninferiority Margin Size

Noninferiority Margin	No. of Respondents (*N* = 106)	No, Reject Shorter Treatment Duration, *n* (%)	Yes, Accept Shorter Treatment Duration, *n* (%)	Overall Chi-Square *P* Value
5%	33	9 (27)	24 (73)	0.003
10%	34	7 (21)	27 (79)	
20%	39	22 (56)	17 (44)	

**Figure 1:**
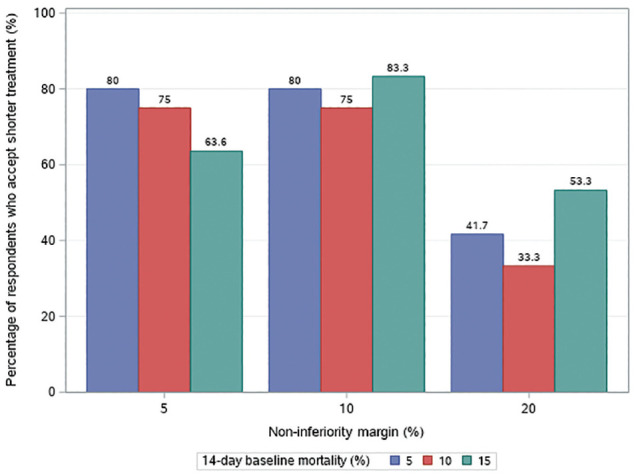
Subgroups of respondents accepting shorter-duration treatment in each noninferiority margin level.

There were no significant differences in the proportions of respondents who would accept shorter-duration treatment among different baseline mortality rates in the 14-d control group (Figure 1). Between specialties, critical care clinicians and pharmacists appeared to be more willing to reject shorter-duration treatment as noninferiority margins increased, but this pattern was not observed among ID physicians. However, this analysis was underpowered, and the overall differences in the proportions who would accept or reject shorter-duration treatment between practice specialties were not significant (Supplemental Table S2).

Logistic regression, adjusted for baseline mortality rates in the control group, indicated that absolute noninferiority margins of 5% and 10% were more likely to be associated with acceptance of shorter durations than if the noninferiority margin was 20% (odds ratio [OR] 3.5, 95% confidence interval [CI]: 1.3–9.6, *P* = 0.013; OR 5.1, 95% CI: 1.8–14.6, *P* = 0.002). The baseline mortality rate in the control group was not a significant independent predictor of the acceptance of shorter treatment (*P* > 0.05).

## Discussion

Using a modified contingent valuation approach to elicit clinician preferences, we found that clinicians are more likely to accept shorter-duration treatment based on noninferior mortality results if noninferiority margins are 5% or 10% than if the margin is 20%. However, nearly half of respondents who reviewed abstracts with a 20% noninferiority margin were still willing to accept shorter-duration treatment.

Acceptance of shorter treatment was not influenced by baseline mortality rates in the control group, suggesting that survey respondents valued relative mortality changes similarly across baseline risks. This contrasts with results from a prior survey that found respondents were willing to accept larger increases in mortality with new treatments when baseline risks were higher.^8^ However, that survey explicitly asked respondents to specify acceptable absolute risk differences in clinical vignettes rather than assess acceptance of noninferior results in mock abstracts. Their respondents were trialists and perhaps were more inclined to consider risk reduction on a relative rather than absolute scale,^8^ whereas we surveyed practicing pediatric clinicians, who may be more inclined to do everything possible to prevent a death in a child as a principle of practice.

We also demonstrated the feasibility of using a modified contingent valuation methodology to elicit acceptance of noninferior mortality from the perspective of end users of the medical literature. The noninferiority margin implies a tradeoff between less effectiveness (i.e., increased mortality) and perceived advantages of a new treatment (i.e., shorter antibiotic exposure).^9^ Researcher-selected noninferiority margins may be pragmatic and influenced by logistical constraints of trial design, so sample size and trial parameters may not always reflect clinicians’ perspectives. Researchers need to use clinically appropriate noninferiority margins and also ensure that the concepts of noninferiority and margin size are comprehensible to a clinical readership. To the best of our knowledge, this is the first application of contingent valuation to understand and elicit willingness to accept study results from the perspective of clinicians. Understanding the impact of decisions made when selecting research study design variables from both the researchers and users’ perspectives could help guide methodology decisions of future noninferiority trial designs and potentially maximize acceptance and facilitate application of trial results to clinical practice.

A strength of this study is the adequate sample size and questionnaire methodology in which we used a modified approach similar to economic contingent valuation. The dichotomous “take it or leave it” question format asking respondents whether they would accept the trial’s noninferior mortality results with a shorter treatment duration was cognitively meaningful and approximates real-life clinical practice decisions. The “take it or leave it” format is a preferred method of elicitation in contingent valuation studies because it is less prone to anchoring biases in comparison with open-ended questions.^5,10,11^ External generalizability was improved by including a multidisciplinary group of clinicians with a wide range of experience across multiple continents.

A limitation of our study was that we presented each respondent with only 1 mock abstract in order to decrease cognitive burden. The acceptance of noninferior mortality results elicited in our study cannot be extrapolated beyond our hypothetical scenario. Another limitation is that only critical care, ID, and intensive care– and ID-focused clinical pharmacists were surveyed, as we wished to focus on critically ill children. Our study was not powered to explore differences by specialist subgroups.

## Conclusions

This study used a novel approach to elicit the acceptance of research design parameters from the perspective of end users of the medical literature. We found that clinicians are more likely to accept shorter treatment durations based on noninferior mortality results when margins are ≤10%, compared with 20%. Yet, nearly half of clinicians would still accept shorter-duration treatment as noninferior with margins as high as 20%. Future research could incorporate additional attributes of research design or clinical conditions and explore how they could also influence acceptance of research results.

## Supplemental Material

sj-docx-1-mdm-10.1177_0272989X221099493 – Supplemental material for Noninferiority Margin Size and Acceptance of Trial Results: Contingent Valuation Survey of Clinician Preferences for Noninferior Mortality Click here for additional data file. Supplemental material, sj-docx-1-mdm-10.1177_0272989X221099493 for Noninferiority Margin Size and Acceptance of Trial Results: Contingent Valuation Survey of Clinician Preferences for Noninferior Mortality by Sandra Pong, Robert A. Fowler, Nicholas Mitsakakis, Srinivas Murthy, Jeffrey M. Pernica, Elaine Gilfoyle, Asha Bowen, Patricia Fontela, Winnie Seto, Michelle Science, James S. Hutchison, Philippe Jouvet, Asgar Rishu and Nick Daneman in Medical Decision Making
