# Supplementary material for: Noninferiority Margin Size and Acceptance of Trial Results: Contingent Valuation Survey of Clinician Preferences for Noninferior Mortality
Source: Med Decis Making. 2022 May 18;42(6):832–6. doi: 10.1177/0272989X221099493 (PMC9277322; doi:10.1177/0272989X221099493)
Supplement: sj-docx-1-mdm-10.1177_0272989X221099493 – Supplemental material for Noninferiority Margin Size and Acceptance of Trial Results: Contingent Valuation Survey of Clinician Preferences for Noninferior Mortality [file sj-docx-1-mdm-10.1177_0272989X221099493.docx]

**APPENDIX**

**Supplement Table 1.** Study parameters for hypothetical abstract scenarios

| Absolute NI margin (%) | 14-day mortality (%) | 7-day mortality (%) | Mortality difference (%) | 95% CI for difference | Sample size |
| --- | --- | --- | --- | --- | --- |
| 5 | 4.8  10  15.2 | 5.2  10.7  16 | 0.4  0.7  0.8 | -3.8 to 4.6  -3.5 to 4.9  -3.3 to 4.9 | 500  900  1300 |
| 10 | 5.6  10.4  15.2 | 6.7  11.2  16.4 | 1.1  0.8  1.2 | -7 to 9.2  -7.7 to 9.3  -7.3 to 9.7 | 180  250  330 |
| 20 | 5.5  10.8  15 | 12.7  16.9  23 | 7.2  6.1  8 | -5.2 to 19.7  -7.2 to 19.5  -3.8 to 19.8 | 110  130  200 |

NI, non-inferiority; CI, confidence interval

**Supplement Table 2.** Acceptance vs. rejection of shorter treatment duration grouped by clinician specialty

| Non-inferiority margin | Specialty | Number of respondents | No, reject shorter treatment duration, n (%) | Yes, accept shorter treatment duration, n (%) | Fisher’s Exact test p-value |
| --- | --- | --- | --- | --- | --- |
| 5% | Critical care  Infectious diseases  Pharmacy | 16  12  5 | 3 (19)  5 (42)  1 (20) | 13 (81)  7 (58)  4 (80) | 0.06 |
| 10% | Critical care  Infectious diseases  Pharmacy | 23  8  3 | 5 (22)  1 (13)  1 (33) | 18 (78)  7 (88)  2 (67) | 0.15 |
| 20% | Critical care  Infectious diseases  Pharmacy | 26  8  5 | 13 (50)  6 (75)  3 (60) | 13 (50)  2 (25)  2 (40) | 0.06 |
